# Protective Effect of Polyphenols Purified from *Mallotus oblongfolius* on Ethanol-Induced Gastric Mucosal Injury by Regulating Nrf2 and MAPKs Pathways

**DOI:** 10.3390/antiox11122452

**Published:** 2022-12-12

**Authors:** Shasha Yu, Zhouwei Duan, Peng Li, Shiping Wang, Lijun Guo, Guanghua Xia, Hui Xie

**Affiliations:** 1 Institute of Agro-Products Processing and Design, Hainan Academy of Agricultural Science, Haikou 571100, China; 2 College of Food Science and Technology, Hainan University, Haikou 570228, China; 3 Sanya Institute, Hainan Academy of Agricultural Sciences, Sanya 572000, China; 4 College of Food Science and Technology, Huazhong Agricultural University, Wuhan 430070, China; 5 College of Feng Xiang, Hainan University, Haikou 570228, China

**Keywords:** *Mallotus oblongifolius* polyphenols (MOP), purification, gastric mucosal injury, oxidative stress, ethanol

## Abstract

*Mallotus oblongifolius* (MO), which is rich in polyphenols, is a characteristic tea resource with medicinal value. In this study, a total of 45 polyphenolic components of MO, including narirutin, isoquercitrin, rutin and digallic acid, were identified by UPLC-Q-TOF/MS analysis. In addition, the gastroprotective effect of *Mallotus oblongifolius* polyphenols (MOP) on ethanol-induced gastric mucosal injury in rats was investigated. The rats received anhydrous ethanol after continuous gavage of MOP or lansoprazole for one week. In addition, the macro- and micro-damage induced by ethanol in the gastric tissue was significantly reduced after MOP pretreatment for one week. Further analysis showed that MOP prevented ethanol-induced acute gastric mucosal injury by increasing the expression of antioxidant enzymes (SOD, CAT, GSH-Px) and decreasing the expression of reactive oxygen species (ROS), lipid oxidation product (MDA) and myeloperoxidase (MPO). Meanwhile, MOP inhibited the phosphorylation of p38/ERK/JNK and promoted the activation of the Nrf2 pathway. These results suggested that MOP may be a promising therapeutic target for the prevention of ethanol-induced gastric mucosal injury by improving oxidative stress, inhibiting the p38/ERK/JNK signaling pathways and activating Nrf2 expression.

## 1. Introduction

The gastric mucosa, which is the mucosa of the inner surface of the stomach, is irregularly wrinkled and has a special gastric protective effect. When an imbalance between attack factors (such as gastric acid, alcohol abuse, nonsteroidal anti-inflammatory drugs) and defense factors (such as prostaglandin, bicarbonate, mucin, mucosal blood flow) occurs in the stomach, the gastric mucosal barrier will be destroyed, resulting in gastric mucosal damage, specifically manifested as gastric mucosal swelling, bleeding, and even gastric ulcer [1]. However, with the accelerated pace of life and the increase in stress in life, alcohol consumption has become a common phenomenon, which is closely related to health problems [2]. Alcohol is a highly corrosive, fat-soluble substance that destroys the normal physiological environment and break the barrier of the gastric mucosa, and is an important factor in causing haemorrhagic gastric mucosal damage [3,4]. Previous studies have shown that lipid peroxidation and oxidative stress induced by alcohol are the cause of gastric mucosa injury [5]. As an invasion factor of the gastric mucosa, oxygen free radicals occupy a vital place in the pathophysiological changes of gastric mucosa injury by causing oxidative damage [6].

Several drugs, including antacids (sodium bicarbonate, aluminium hydroxide), proton pump inhibitors (omeprazole, lansoprazole), antibiotics (Helicobacter pylori inhibition) and H_2_ receptor antagonists (cimetidine, ranitidine), have been used to treat and ameliorate gastric mucosal injury. However, there is growing evidence that these drugs have limited effects and may cause side effects in many aspects when taken over a long period of time [7,8,9]. Therefore, it is vital to find efficient, safe and economical alternative drugs to avoid the suffering of gastric mucosa injury. Natural products have become a hot topic of scientific research as possible alternatives to stomach protection. Among these, polyphenols have attracted particular attention and have been shown to exert a protective effect in the stomach by inhibiting oxidative stress.

*Mallotus oblongifolius* (MO) is a characteristic substitute for tea beverages in Hainan, China [10]. It is locally known as “glossy ganoderma grass” with important medicinal and economic value [11]. It is reported to have a variety of beneficial effects, including antioxidant, antibiosis, reducing blood pressure, nourishing the stomach and so on. Most of these effects are related to the active substances contained in MO, especially polyphenols. Recent studies have found that gallic acid has a gastric protective effect on ethanol-induced gastric mucosal injury in rats [12]. However, the composition analysis of *Mallotus oblongifolius* polyphenols (MOP) and its protective mechanism against gastric injury have not been investigated. Notably, MO is a traditional tea with medicinal value, but its value has not been fully exploited due to limited research and development. In this study, the purification method of MOP was optimized, the main components of MOP were identified by UPLC-Q-TOF/MS analysis and the potential mechanism of preventing ethanol-induced gastric mucosal injury in rats was explored by biomolecular detection, which provides reference values for the in-depth development of MO resources.

## 2. Materials and Methods

### 2.1. Materials and Chemicals 

The MO was obtained from Hainan Academy of Agricultural Sciences (Hainan, China). The absolute alcohol was purchased from Xilong Science Co., Ltd. (Shantou, China). The enzyme-linked immunoassay assay (ELISA) kits for reactive oxygen species (ROS) and myeloperoxidase (MPO) were purchased from Shanghai X-Y Biotechnology Co., Ltd. (Shanghai, China). The assay kits for malondialdehyde (MDA), superoxide dismutase (SOD), glutathione peroxidase (GSH-Px) and catalase (CAT) were purchased from the Nanjing Jiancheng Bioengineering Institute (Nanjing, Jiangsu, China). The primers (Nrf2, Keap1, HO-1, p38, JNK and ERK) were obtained from Sangon Biotech Co., Ltd. (Shanghai, China). The antibodies (Nrf2: ab92946, Keap1: #8047S, HO-1: ab189491, p38: ab4822, p-p38: ab4822, JNK: ab179461, p-JNK: ab76572, ERK: ab184699 and p-ERK: ab201015) were purchased from Abcam (Cambridge, UK) and Cell Signaling Technology (Boston, MA, USA).

### 2.2. Extraction of Crude MOP

The methods used to prepare the polyphenol extracts were slightly improved, according to a previous study [13]. The MO powder (100 g) was extracted with 50% ethanol (1500 mL) at 55 °C for 2 h. Next, the solid-liquid mixture was filtered to obtain the MO extract. The filtrates were then evaporated at 55 °C by a rotary evaporator (RE52AA). Finally, the crude MOP was obtained by vacuum freeze-drying.

### 2.3. Purification of MOP

#### 2.3.1. Static Adsorption and Desorption Kinetics

Following pretreatment, 5 g of NKA−9 macroporous resin was placed into a 250 mL triangular flask, and 100 mL of crude polyphenol extraction solution (5 mg/mL) was added. Adsorption equilibrium was achieved by shaking for 12 h on a shaker (30 °C, 180 r/min). The Folin-Ciocalteu method was used to determine the mass concentration of the polyphenols after adsorption, each at 0.5 h. Once adsorption equilibrium was achieved, the solution was desorbed with 100 mL 60% (*V*/*V*) ethanol solution on a shaker (30 °C, 180 r/min). The mass concentration of the polyphenols in the solution was measured, each at 0.2 h. 

#### 2.3.2. Effects of Different Factors on Adsorption and Desorption 

As described in Section 2.3.1, the macroporous resin was mixed with the crude extract, and the polyphenol concentration (C_e_, mg/mL) in the filtrate was determined after adsorption. Then, the macroporous resin was desorbed with ethanol solution on a shaker with the same rotational speed, and the concentration of polyphenols (C_d_, mg/mL) in the filtrate was determined after desorption. The type of resin, concentration of crude extract and concentration of ethanol in the purification process were determined according to the adsorption and desorption effect. The following formulas were used to calculate the adsorption capacity, adsorption rate, resolution rate.

The adsorption rate: (%) = (C_0_ − C_e_)/C_0_ × 100

The desorption rate: (%) = (C_d_ × V_d_)/[(C_0_ − C_e_) ×V_0_] × 100

The adsorption quantity: (mg/g) = (C_0_ − C_e_) × V_0_/M

where C_0_ and C_e_ are the mass concentration of the polyphenol at the beginning and adsorption equilibrium, respectively (mg/mL); C_d_ is the mass concentration of the polyphenol in the desorption solution (mg/mL); V_0_ is the initial volume of the crude extract; V_d_ is the volume of the ethanol desorption solution (mL); and M is the weight of macroporous resin (g).

#### 2.3.3. Dynamic Adsorption and Desorption

The glass column (16 mm × 500 mm) was filled with pretreated NKA−9 resin for dynamic adsorption and desorption experiments. The sample solution flowed through the glass column at different flow rates (1.0, 1.5, 2.0, 2.5, 3.0, 3.5 mL/min). The adsorption rate was calculated according to the concentration of polyphenol in the effluent sample solution. After the adsorption was complete, the glass column was eluted with 60% (*V*/*V*) alcohol solution at different flow velocities (1.0, 1.5, 2.0, 2.5, 3.0 mL/min). After desorption, the mass concentration of the polyphenols in the effluent solution was ascertained, and the desorption rate of the resin was calculated. The optimum flow rates of the sample solution and elution were determined according to the adsorption rate and desorption rate, respectively.

The sample solution was permitted to flow through the glass column at a rate of 1.5 mL/min. The effluent solution was collected. One tube was 9 mL, and 10 tubes were a bed volume (BV). The dynamic adsorption curve was drawn with BV as the abscissa and polyphenol concentration in the effluent as the ordinate. After the dynamic adsorption was complete, 60% (*V*/*V*) ethanol was used as the eluent at a flow rate of 2.0 mg/mL. The mass concentration of the polyphenols in the effluent solution was determined every 0.2 BV, and the dynamic elution curve was drawn with the above method.

### 2.4. Method for Composition Analysis of MOP

#### 2.4.1. UPLC-Q-TOF/MS Analysis

The sample was analyzed by using an Acquity UPLC I-Class System high-performance fluid chromatograph equipped with a Xevo G2-XS QTOF mass spectrometer, coupled with an ACQUITY UPLC^®^ HSS T3 column (100 mm × 2.1 mm, 1.8 μm) maintained at 35 °C. The purification of the polyphenols was redissolved in 70% (*v*/*v*) ethanol and then filtered with a 0.22 μm nylon filter. The ionization conditions were set as follows: collision energy ramp 30–60 eV; cone voltage of 40 V, trap collision energy of 6.0 V; ramp collision energy from 20 to 30 V; capillary voltage of 2.5 kV; sample temperature 20 °C; source temperature of 120 °C; desolvation temperature of 450 °C; cone gas flow of 50 L/h; and desolvation gas flow of 900 L/h. The scanning time of the whole detection process is 1.5 s. Furthermore, leucine-enkephalin (*m*/*z* 556.2771, [M + H]+) solution at a concentration of 200 ng/μL, with a flow rate of 10 μL/min, was used as the external reference to ensure accuracy during the analysis. Elution was performed with a mobile phase of A (0.1% formic acid) and B (acetonitrile) under a gradient program: 0–1 min, 5% B; 1–2 min, 5–10% B; 2–4 min, 10–15% B; 4–16 min, 15% B; 16–21 min, 15–17% B; 21–30 min, 17% B; 30–32 min, 17–22% B; 32–34 min, 22–30% B; 34–36 min, 30–32% B; 36–38 min, 32–35% B; 38–40 min, 35–40% B; 40–42 min, 40–45% B; 42–44 min, 45–50% B; 44–46 min, 50–60% B; 46–56 min, 60–70% B; 56–62 min, 70–80% B; 62–64 min, 80–95% B; 64–67 min, 95% B; 67–67.1 min, 95–5% B; 67.1–70 min, 5% B. The flow velocity was 0.3 mL/min, and the sample volume was 3 μL. In positive ion mode, the mass range was set to *m*/*z* 100–1500 Da. The data were recorded by the TOF MSE (MS at Elevated Fragmentation Energy) model.

#### 2.4.2. Chemical Composition Analysis

The UPLC-Q-TOF/MS data acquisition from MasslCIx software (version 4.1, Waters Corporation, Milford, MA, USA) was imported to Progenesis QI software (version 2.0, Waters Corporation) for data handling, involving peak detection, alignment and normalization. The compound information, scanned by UPLC-Q-TOF/MS, was compared with the database by UNIFI software. The analysis conditions were set as follows: mass error ≤ 3 mDa, chromatographic peak extraction time 0–70 min, positive ion mode adduct ions +H, +Na.

### 2.5. Animals and Experimental Design

#### 2.5.1. Arrangement of Animals and Induction of the Model

Seven-week-old male Sprague-Dawley rats (200 ± 20 g) were purchased from Changsha Tianqin Biotechnology Co., Ltd. (Changsha, China, Certificate number: SCXK (Xiang) 2019-0013). All rats were fed a standard diet and were given free access to water. They acclimated for a week before the experiment; they were placed in a room with conditions of 23 °C and 12 h day/night cycle [14,15]. All animal procedures were executed on the basis of the National Guidelines for Experimental Animal Care and Use and were permitted by the ethical committee of experimental animal care of Hainan University (HNDX2020072).

After the seven-day adaptation period, all of the rats were distributed into 6 groups at random (n = 10): the normal control group (NC) and ulcer control group (UC) were treated with vehicle (5% Tween-80); the lansoprazole group (LAN) was treated with 30 mg/kg bw of lansoprazole; the low-dose group of MOP (MOP-L), median-dose group of MOP (MOP-M) and high-dose group of MOP (MOP-H). MOP-L, MOP-M and MOP-H were treated with 50 mg/kg bw, 100 mg/kg bw and 200 mg/kg bw MOP, respectively, which were dissolved in 5% Tween-80. After continuous gavage for a week, all of the rats were fasted but kept in water for 24 h. On the eighth day, with the exception of the NC group, all groups were given 5 mL/kg anhydrous ethanol by gavage. Four hours later, the rats were anesthetized with 10% chloral hydras and sacrificed by cervical dislocation [16,17]. The required samples were collected and preserved.

#### 2.5.2. Sample Collection and Preservation

The collected blood were centrifuged at 4000× *g* for 10 min to obtain serum. The serum was divided into several portions and stored at −80 °C for next experiment. At the same time, the stomach was cut along a larger bend with scissors and rinsed with cold normal saline. Then, the stomach tissue was photographed after blotting the rest of the saline with filter paper. Thereafter, the gastric tissue was randomly divided into two parts. One part was immersed in 4% paraformaldehyde, and the other part was collected in a freezer tube and saved in a −80 °C refrigerator.

#### 2.5.3. Macroscopic and Microscopic Injury Assessment of Gastric Tissue

Image J (1.47v, National Institutes of Health, Bethesda, MD, USA) was used to analyze the photos of the stomach tissue. The ulcer area (UA, mm^2^) was measured, and the ulcer index was evaluated on account of the formula below:

UI = 10/X

where X is the ratio of the total stomach mucosa area to the total ulcer area

In addition, the stomach tissues, soaked in 4% paraformaldehyde, were embedded in paraffin and sliced. These sections were used for hematoxylin-eosin staining (H&E) to observe and analyze the microscopic lesions of gastric tissue [18,19].

#### 2.5.4. Determination of Oxidative Stress Cytokines

The gastric tissues were mixed with cold phosphate buffer saline (PBS) to prepare 10% homogenate, which was centrifuged at 4000× *g* at 4 °C for 10 min. The ROS (XYR9212641) and MPO (XY9R0142) levels in the stomach tissues were detected by ELISA kits. The expression of MDA (A003-1), SOD (A001-3), GSH-Px (A005-1) and CAT (A007-1-1) in the stomach tissues was determined by biochemical kits. All kits are operated in accordance with the manufacturer’s instructions.

#### 2.5.5. Real-Time Quantitative PCR

Total RNA extraction was performed from the gastric tissues using the RNA prep Pure Tissue Kit (Tiangen, Beijing, China). In brief, 10–20 mg of stomach tissues was mixed with 300 μL RL lysate and fully ground. Proteinase K was added, and the mixture was treated at 56 °C for 10–20 min. After centrifugation, they were treated with anhydrous ethanol, Buffer RW1, DNase I working solution and Buffer RW, respectively. Finally, 30–100 μL of RNase-Free ddH_2_O was added to obtain total RNA samples of gastric tissues. The total RNA extracted was mixed with 5× FastKing-RT SuperMix and RNase-Free ddH_2_O to establish a 20 μL reaction system by FastKing gDNA Dispelling RT SuperMix Kit (Tiangen, Beijing, China). Another 20 μL reaction system (10 μL 2× SuperReal PreMix Plus, 0.6 μL forward primer, 0.6 μL reverse primer, 1.5 μL cDNA, 7.3 μL RNase-Free ddH2O) was established to complete RT-PCR on the CFX Connect RT-PCR platform (Bio-Rad, Hercules, CA, USA). PCR amplification was performed using the following cycling conditions: activation of HotStar TaqDNA polymerase and initial denaturation at 95 °C for 15 min, and 40 cycles of PCR reactions at 95 °C, 60 °C and 72 °C. All kits were operated according to the manufacturer’s instructions. Relative gene expression was standardized to GAPDH by the 2^−ΔΔCT^ method [20,21]. The relevant primers information is supplemented in the additional document (Supplementary Table S1).

#### 2.5.6. Western Blot Analysis

RIPA lysate buffer (Servicebio, Wuhan, China) was used to separate protein from gastric tissue, and the protein concentration was measured by a BCA kit (Servicebio, Wuhan, China). Prepared protein samples were separated by gel electrophoresis and transferred to solid phase carrier (PVDF) membranes, which were incubated with special antibodies and washed with TBST. After that, the membranes were soaked in ECL chemiluminescence solution to detect the target protein blots which were visualized by ImageQuant LAS4000mini system (GE, Boston, MA, USA). During the whole process, β-actin was treated as a control protein [22,23].

### 2.6. Statistical Analysis

The experimental data are expressed as the mean values ± standard deviation (SD) and were analyzed with SPSS 25.0 software (IBM Inc., Armonk, New York, NY, USA) or GraphPad Prism 8 (San Diego, CA, USA). One-way analysis of variance (ANOVA) was utilized to evaluate differences among groups. When the *p* value was less than 0.05, the difference was considered statistically significant.

## 3. Results

### 3.1. Static Adsorption and Desorption

#### 3.1.1. Static Adsorption and Desorption Kinetics

**Figure 1:**
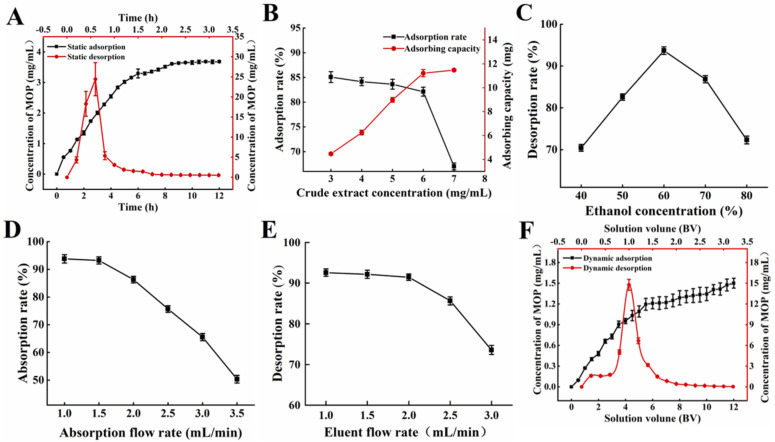
Adsorption and desorption capacities of NKA−9 resin for polyphenols from MO. (**A**) Kinetics for static adsorption and desorption on NKA−9 resin for polyphenols from MO. (**B**) Effect of concentration of initial MOP on adsorption rate on NKA−9 resin for polyphenols from MO. (**C**) Effect of eluent concentration on desorption rare on NKA−9 resin for polyphenols from MO. (**D**) Effect of adsorption flow rate on adsorption rate on NKA−9 resin for polyphenols from MO. (**E**) Effect of eluent flow rate on desorption rate on NKA−9 resin for polyphenols from MO. (**F**) Kinetics for dynamic adsorption and desorption on NKA−9 resin for polyphenols from MO.

The static adsorption and desorption curves of NKA−9 resin on the polyphenols of MO leaves are shown in Figure 1A. NKA−9 macroporous resin was used for the rapid adsorption of the polyphenols from the MO leaves. The adsorption capacity increased sharply within 6 h. After 8.5 h, the concentration of polyphenols did not change significantly, showing that the adsorption of the polyphenols had reached equilibrium. It could be seen from the desorption curve that the 60% (*v*/*v*) ethanol solution had a good desorption effect on polyphenols adsorbed by resin, and the desorption rate increased rapidly in the initial stage (within 0.6 h) and reached equilibrium after 2 h.

#### 3.1.2. Influence of Resin Type on Adsorption and Desorption Effect

The adsorption and dissolution of polyphenols are closely related to the spatial structure, polarity and water content of macroporous resin. As shown in Supplementary Table S2, among the five kinds of macroporous resins, the highest adsorption rate on MOP was D201 with 56.07%, followed by NKA−9 with 54.65%. However, the highest desorption rate on MOP was NKA−9, which was up to 92.35%. Therefore, NKA−9 was used to adsorb and desorb MOP. This was because the polarity, structure and physicochemical properties of the NKA−9 resin were more conducive to the adsorption of MOP. Van der Waals forces and hydrogen bonds were the main forces between the two and were weaker and more easily broken than ionic bonds, favoring elution.

#### 3.1.3. Influence of Concentration of Initial MOP on Adsorption Effect

The influence of the initial MOP concentration on the adsorption effect is demonstrated in Figure 1B. When the initial MOP concentration was 6 mg/mL, a high adsorption rate and adsorption capacity were observed. The adsorption rate decreased significantly, while the adsorption amount tended to saturate if the initial MOP concentration surpassed 6 mg/mL. When the concentration of the crude extract was lower than 6 mg/mL, the NKA−9 resin did not reach saturation. With the increase in the crude extract concentration, the contact amount between the resin and polyphenols, per unit surface area, was augmented, and the adsorption amount increased. Nevertheless, the adsorption rate decreased slightly because of the reduction in free groups on the resin surface. When the concentration of the crude extract exceeded 6 mg/mL, the mass transfer rate slowed down with the gradual saturation of groups on the resin surface, and some polyphenols flowed out without being adsorbed, resulting in a decrease in the adsorption rate. The resin gradually reached saturation adsorption, and the adsorption amount changed very little.

#### 3.1.4. Influence of Eluent Concentration on Desorption Effect

The effect of ethanol concentration on NKA−9 resin desorption can be seen in Figure 1C. The desorption rate of the NKA−9 resin increased with the eluent concentration, from 40% to 60%. The optimal desorption rate of 93.69% was observed at an eluent concentration of 60%. As the ethanol increased, the desorption rate began to decrease. The ethanol concentration was related to its polarity, which could change the molecular forces between the polyphenols and macroporous resins. In addition, the solubility of the polyphenols was altered in different concentrations of ethanol. The polyphenols were not easily dissolved in low concentrations of ethanol. However, the ethanol concentration was too high, and some impurities were also removed. The above two factors led to a great influence of ethanol concentration on the resin desorption.

### 3.2. Dynamic Adsorption and Desorption

#### 3.2.1. Influence of Adsorption Flow Rate on Adsorption and Desorption Effect

The flow rate of the sample solution affected the reaction between the solute and NKA−9 resin and further affected the adsorption and desorption effect of the resin. Figure 1D demonstrated that the adsorption rate of the resin decreased with the increasing flow rate of the sample solution. As the sample flow rate increased, the time that the crude extract remained in the column decreased, and part of the MOP was not adsorbed by the resin in time and then flowed out of the column [24]. It is worth noting that the adsorption rate of the NKA−9 resin on the polyphenols decreased by 0.56% at a 1.5 mL/min flow rate compared with that at a 1.0 mL/min flow rate, and the difference was not significant; however, the sample loading amount of crude extract per unit time increased by 0.5 times. Therefore, the optimal sample flow rate was 1.5 mL/min. Moreover, the desorption rate gradually decreased with the increasing flow rate of the eluent (Figure 1E). When the elution flow rate was higher than 1 mL/min, the target substance in the resin was not desorbed by the eluent in time, and the elution was incomplete. A flow rate of 1.0 mL/min was conducive to the desorption of the polyphenols from the resin. This may be due to ethanol entering the resin micropores at low flow rates, allowing the polyphenols to dissolve and elute more thoroughly [25]. However, a slow flow rate would prolong the purification cycle. In comparison to the elution flow rate of 1.0 mL/min, the desorption rate of the resin on the polyphenols only decreased by 1.12% at 2.0 mL/min. It is evident tht there was no significant difference; however, the time taken to desorb the resin with the same volume of ethanol was halved. Hence, 2.0 mL/min was chosen as the most appropriate elution velocity.

#### 3.2.2. Dynamic Adsorption and Desorption Kinetics

The sample solution was pressed into a glass column filled with NKA−9 resin through a peristaltic pump. The dynamic adsorption curve is shown in Figure 1F. When the inflow volume was 3.0 BV, the polyphenol concentration of the effluent solution was 0.729 mg/mL, which was approximately 46% of the initial sample, indicating that most of the polyphenols in the sample were adsorbed by NKA−9 resin. The injection volume was 11.5 BV, the concentration of polyphenols in the effluent solution was 1.475 mg/mL, which was close to the concentration of polyphenols in the initial sample. As the injection volume continued to increase, the polyphenol concentration of the effluent solution tended to be stable. The mass concentration of polyphenols in the effluent solution was close to that of the initial sample, indicating that the resin was saturated by adsorption. Thus, the saturated adsorption of the NKA−9 resin was achieved at 11.5 BV. Furthermore, Figure 1F also shows the results of the dynamic desorption kinetics. The 0–2.0 BV effluent solution contained most of the desorbed polyphenols. Subsequently, the concentration of polyphenols in the effluent solution decreased rapidly with the increasing ethanol dosage. When the consumption of ethanol was 2.8 BV, the concentration of polyphenols in the effluent solution was only 0.099 μg/mL, which was approximately 1/16 of the initial sample solution. At this point, the polyphenol concentration in the effluent solution tended to be stable, indicating that the adsorbed polyphenols were basically desorbed completely.

### 3.3. MOP Composition Analysis

#### Polyphenol Compositions of MO

**Figure 2:**
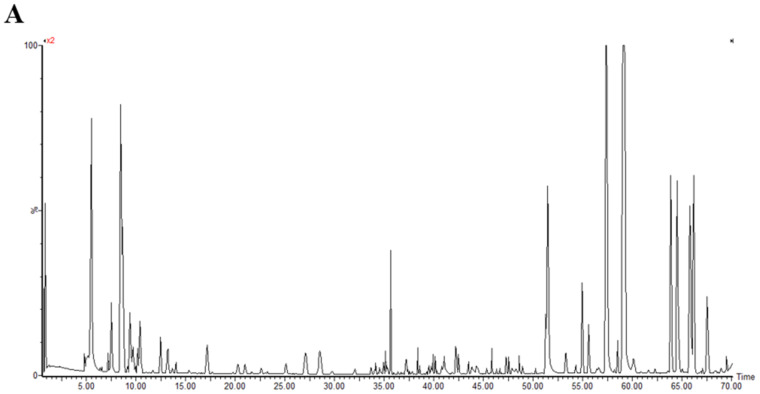

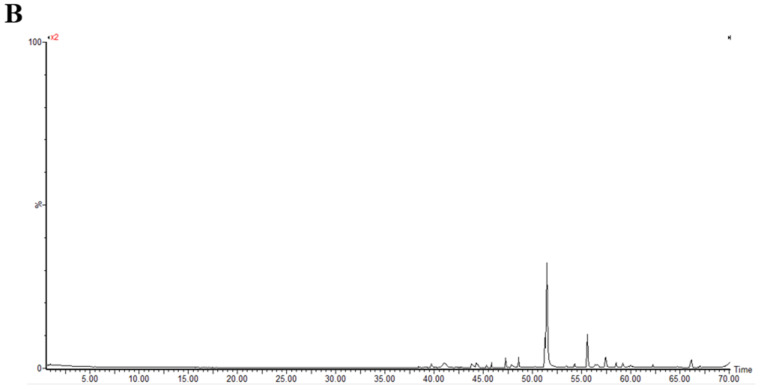
The base peak chromatograms of purified polyphenols from Mallotus oblongifolius (**A**) and the control of acetonitrile (**B**) by UPLC-Q-TOF/MS analysis under positive ion mode.

With the verified UPLC-QTOF/MS means and UNIFI software, a total of 50 polyphenol components were identified in MO. All of the compounds were numbered according to their order of elution (Figure 2). The fragmentation details of 50 compounds are shown in Supplementary Table S3. According to the proposed tactics and comparison of collected data with existing publications, as well as the fragmentation pattern of different components generalized by former studies, the main compositions of the polyphenols in MO were narirutin, isoquercitrin, rutin, digallic acid, vitexin, D-catechin, quercetin, luteolin, kaempferol, myricetin (Supplementary Table S3). However, the components of Nos. 43, 45, 47, 48 and 50 were undefined compared to the database and required further study.

### 3.4. Macroscopic and Microscopic Analysis of Gastric Tissue

**Figure 3:**
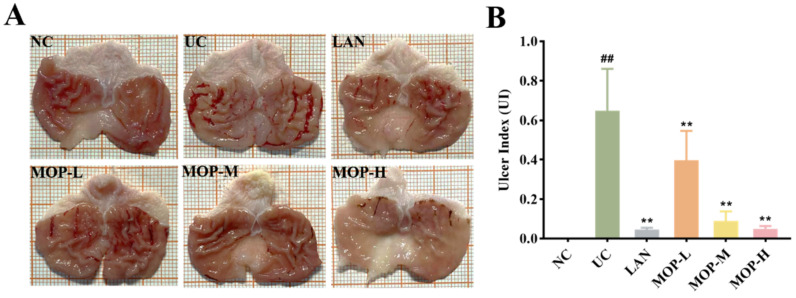

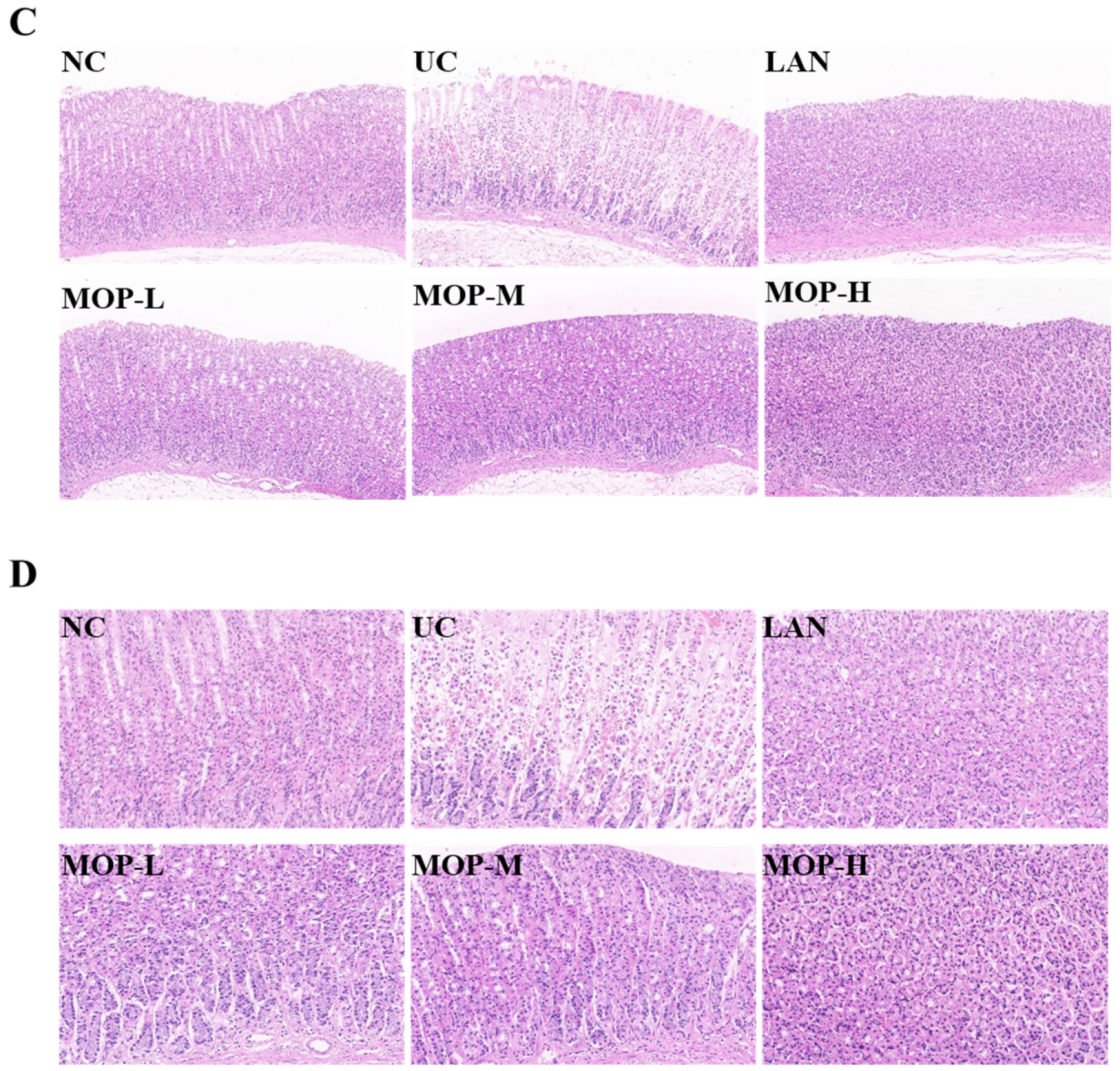
Macroscopic and microscopic injury of gastric mucosal of rats in all experimental groups (n = 10). (**A**) Representative macroscopic images of stomach tissue. (**B**) Effects of MOP on the ulcer index of ethanol-induced gastric mucosal injury. (**C**) Representative histological morphology of stomach tissues (magnification 200×). (**D**) Representative histological morphology of stomach tissues (magnification 400×). # and * denotes significant differences (n = 6). ## *p* < 0.01 vs. NC group; ** *p* < 0.01 vs. UC group.

The macroscopic structure of the gastric tissue showed no tissue lesions in the NC group, while haemorrhagic lesions and elongated strip injuries appeared in the model group (Figure 3A). Lansoprazole or MOP pretreatment attenuated the gastric lesions caused by ethanol, and the effect of MOP at a high dose was improved. Similar results were obtained for the ulcer index (Figure 3B). Compared with the NC group, the ulcer index was significantly increased in the ethanol treatment group, while MOP or LAN pretreatment significantly improved this phenomenon. Meanwhile, the HE results showed that the gastric tissue had areas of necrosis, owing to intragastric alcohol administration, with shedding of many gastric glandular cells, cell nuclei fragmentation or dissolution and a small amount of bleeding, accompanied by lymphocyte infiltration, severe oedema in the submucosa and loose connective tissue arrangement (Figure 3C,D). After MOP or lansoprazole intervention in advance, the microscopic pathological injury of the gastric tissue was significantly improved.

### 3.5. Oxidative Stress Factor Levels

**Figure 4:**
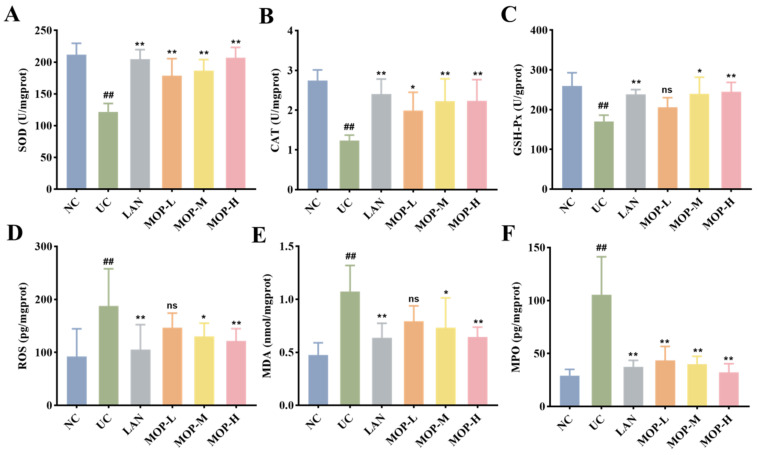
Effects of MOP on levels of SOD (**A**), CAT (**B**), GSH-Px (**C**), ROS (**D**), MDA (**E**), MPO (**F**) in stomach tissue of rats in vitro. # and * denotes significant differences (n = 6). ## *p* < 0.01 vs. NC group; * *p* < 0.05, ** *p* < 0.01 vs. UC group. ‘ns’ denotes no significant difference.

Compared with the NC group, the levels of SOD, CAT and GSH-Px in the UC group were remarkably diminished (*p* < 0.05, Figure 4A–C). Conversely, the SOD level, CAT level and GSH-Px level were increased to different degrees in the positive control LAN group, MOP-L group, MOP-M group and MOP-H group. The ROS and MDA activities showed opposite trends to antioxidant enzyme activities (Figure 4D–E). Compared with the NC group, the activity of ROS, MPO and MDA significantly increased in the gastric tissue of the UC group, and the LAN group and MOP groups at three doses were able to reduce their activity to different degrees (*p* < 0.05).

### 3.6. Effect of MOP on the MAPK and Nrf2 Signaling Pathways

**Figure 5:**
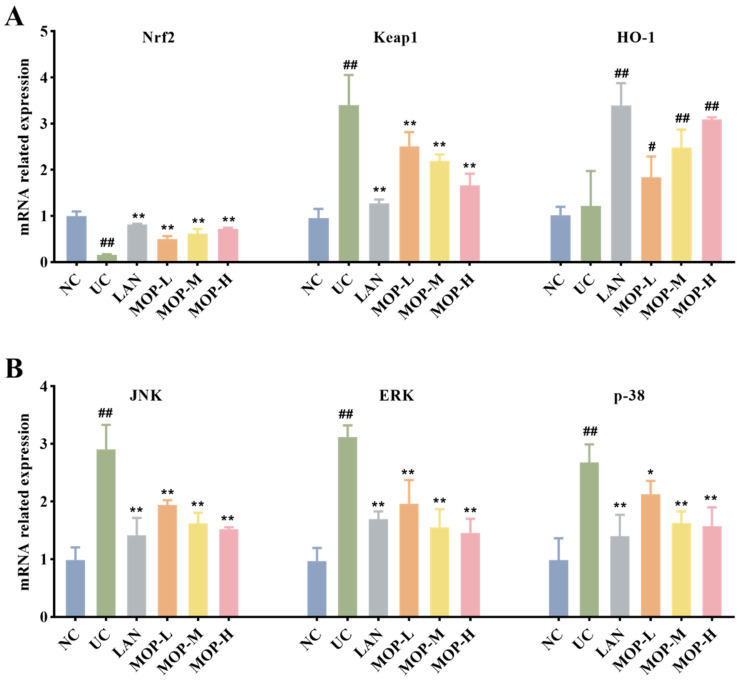
mRNA expression levels of Nrf2, HO-1 and Keap1 (**A**), p38, JNK and ERK (**B**) in stomach tissue of MOP pretreated ethanol-induced gastric mucosal injury. All experiments were performed in triplicate (n = 3) to determine the repeatability. # and * denotes significant differences. # *p* < 0.05, ## *p* < 0.01 vs. NC group; * *p* < 0.05, ** *p* < 0.01 vs. UC group.

**Figure 6:**
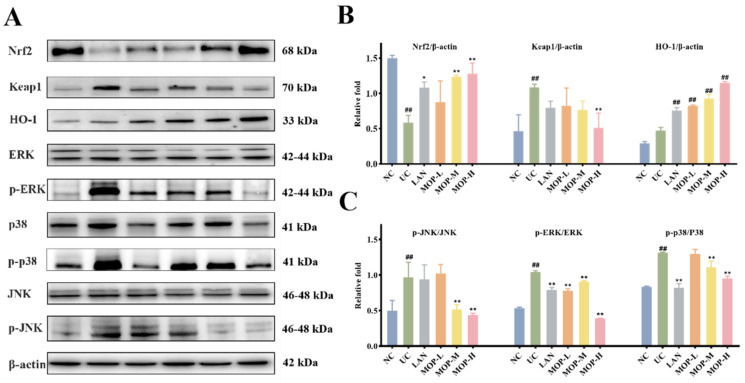
MOP attenuated ethanol-induced gastric mucosal injury by activating Nrf2/HO-1 and inhibiting MAPKs pathway. The western blot bands (**A**) and relative intensities of (**B**) Nrf2, Keap1 and HO-1, (**C**) p-JNK, p-ERK and p-p38. All experiments were performed in triplicate (n = 3) to determine the repeatability. # and * denotes significant differences. ## *p* < 0.01 vs. NC group; * *p* < 0.05, ** *p* < 0.01 vs. UC group.

To confirm whether the Nrf2 pathway protects the gastric mucosa, the gene and protein expression levels of Keap1, Nrf2 and HO-1 were investigated. The level of Keap1 in the gastric stomach was significantly increased only in the alcohol treatment group, while the expression of Keap1 was observably decreased in a dose-dependent manner in the polyphenol pretreatment groups (Figure 5A and Figure 6A). Moreover, compared with the normal group, ethanol gavage resulted in an obvious decrease in Nrf2 but had little effect on HO-1 expression. In contrast, the gene and protein levels of Nrf2 and HO-1 were significantly upregulated by MOP pretreatment.

Furthermore, the MAPK signaling cascade is a major stress signaling pathway related to oxidative stress [26]. Next, in order to further verify the therapeutic target of MOP on ethanol-induced gastric mucosal injury, the effects of MOP on ethanol-induced gastric mucosal injury and the expression of JNK, ERK, and p38 were evaluated. The results showed that ethanol treatment alone significantly stimulated the expression of JNK, ERK and p38 (Figure 5B and Figure 6B). However, pregavage of MOP apparently reversed this change. Among them, the MOP high-dose group had the best effect.

## 4. Discussion

Polyphenols are natural active ingredients in plants and have potential antioxidant properties [27]. In this study, the purification method of polyphenols from MO was investigated to determine the influencing factors, such as resin type, crude extract concentration and flow rate, as well as eluent concentration and flow rate during the purification process. UPLC-Q-TOF/MS analysis demonstrated that the main composition of MOP was rutin, gallogen, vitexin and catechin. In addition, we investigated the effect of MOP pretreatment on ethanol-induced gastric mucosal damage and found that it exhibited good intervention.

Previous studies have shown that ethanol is a key factor in causing gastric mucosal injury and upper gastrointestinal bleeding [28,29]. Usually, an ethanol induced gastric mucosa injury model is used to study the pathogenesis of gastric ulcers and the intervention effect of active substances or drugs [30]. This caused infiltration of inflammatory cell and production of large amounts of ROS, which further triggered oxidative stress [31,32]. In this study, it was found that oxidative stress damage was particularly severe in the ethanol treatment group (Figure 3, Figure 4, Figure 5 and Figure 6). These phenomena are consistent with previous studies and demonstrates that ethanol as an oxidative ulcer inducer is persuasive in evaluating the antioxidant role of MOP in gastric protection. Ethanol treatment caused a series of complex morphological and functional changes in the gastric tissue [33]. From a macro and micro perspective, MOP pretreatment effectively alleviated ethanol-induced gastric mucosa injury. Compared with the ethanol treatment group, the lesion area and ulcer index of gastric mucosa in the MOP treatment groups were significantly reduced in a dose-dependent manner. At the same time, this result was supported by histopathological changes, with the alleviation of symptoms such as bleeding, submucosal edema and inflammatory cell infiltration. These results were consistent with previous studies evaluating the gastric protective effect of different synthetic compounds and confirmed the gastric protective effect of MOP on ethanol-induced gastric mucosal injury.

The pathological changes to gastric mucosa injuries are closely related to oxidative stress. Ethanol is first metabolized in the stomach by alcohol dehydrogenase to produce a large amount of acetaldehyde, which generates oxygen free radicals under the action of xanthine oxidase [34]. Excessive oxygen free radicals notably enhance oxidation and lead to an oxidative stress state [35]. It has been reported that oxidative stress plays a key role in alcohol-induced gastric mucosal injury [36]. Alcohol causes the superabundant production of ROS in the gastric mucosa and leads to oxidative stress [37]. Despite the destructive aftermath of the overproduction of ROS, the defense systems of tissues and cells respond to oxidation-related cell and tissue damage. An excess of ROS consumes more antioxidant enzymes and nonenzymatic substances (such as SOD, CAT, GSH-Px) and destroys the antioxidant system of the gastric tissue, resulting in excessive lipid oxidation products (such as MDA) [38]. MDA cross-linked with biological macromolecules in vivo and further destroyed the structure and function of proteins. To some extent, the MDA reflected the damage degree of tissue oxidation. Studies have also shown that MOP catalyzed hydrogen peroxide to produce ROS [39]. Thus, these indices reflected the relationship between oxidative stress and gastric tissue injury to a certain extent. In this study, it was found that alcohol treatment significantly increased the levels of ROS, MPO and MDA, while markedly decreasing the levels of SOD, CAT and GSH-Px (*p* < 0.05, Figure 4A–E). Importantly, MOP intervention inhibited the above change, manifesting its antioxidant potential. These results confirmed that alcohol induced severe oxidative stress and gastric injury, and that MOP pretreatment effectively regulated the state of oxidative stress in gastric tissue.

In addition to causing pathological processes, ROS also plays an important role in cell signal transduction and regulation as second messengers, indirectly participating in immune responses or other cellular protective processes. The Keap1/Nrf2/HO-1 signaling pathway was activated during antioxidative damage processes in cells. Nrf2, belonging to the NF-E2 family, has been reported to play a crucial role in various kinds of cell protection mechanisms [40]. Under normal physiological conditions, Nrf2 is inactivated in the cytoplasm due to coupling with Keap1. With a large increase in ROS, the conformation of Keap1 is changed, and Nrf2 is transferred to the nucleus, where it combines with ARE (antioxidant reaction element) and activates the downstream factors [41]. In particular, HO-1, which is mainly activated by Nrf2, degrades heme and is an important enzyme for reversing oxidative stress damage [42]. HO-1 degrades heme to CO, biliverdin and Fe^2+^ and is an essential mediator of the antioxidant effect of Nrf2 [43]. It was reported that the enhanced expression of HO-1 had a protective effect on gastric mucosa in alcohol-induced gastric mucosa injury [6]. Our results showed that pretreatment with MOP enhanced the expression of Nrf2 and HO-1 and decreased the expression of Keap1. Thus, Keap1/Nrf2/HO-1 is a potential mechanism for antioxidative damage to gastric mucosa. This result suggested that MOP activated the Nrf2/HO-1 signaling pathway to protect the gastric mucosa.

Notably, MAPK carries signals from the cell surface to the nucleus, which are activated by oxidative stress and various proinflammatory cytokines [44]. It is widely involved in various kinds of endocellular signaling processes and plays a vital role in the activation and expression regulation of the Nrf2/HO-1 pathway [45]. JNK, ERK and p38 are the most studied among MAPK. The inhibition of p38 phosphorylation prevented the Keap1 degradation and nuclear translocation of Nrf2 and subsequently upregulated the induction of HO-1 expression [46]. At present, the regulatory mechanism of ERK and JNK on the Nrf2/HO-1 signaling pathway is still not completely clear. However, phosphorylation of ERK1/2 was reported to significantly reduce the quercetin-induced expression of Nrf2 and HO-1 [47]. Studies have also shown that the downregulation of JNK phosphorylation increased HO-1 expression and contributed to positive intervention in oxidative stress-related diseases [48,49]. Figure 5 and Figure 6 showed that MOP pretreatment significantly inhibited the phosphorylation of JNK, ERK and p38 induced by ethanol treatment alone. These results suggest that MOP may mediate HO-1 expression by inhibiting JNK, ERK and p38 activation. In addition, many studies have shown that MAPKs are also involved in inflammation. The activation of MAPK regulated the expression of inflammatory factors and indirectly activated the downstream NF-κB pathway, which plays an important role in the process of inflammation and immune response. Therefore, the specific mechanism of MAPK-mediated HO-1 expression and its regulation of the inflammatory response in ethanol-induced gastric mucosal injury still need to be further study.

## 5. Conclusions

In this study, the extraction and purification process parameters of MOP were optimized and its preventive effect on ethanol-induced gastric mucosal damage was investigated. A total of 50 polyphenolic components were identified by UPLC-Q-TOF/MS analysis. In addition, the main components of MOP included narirutin, isoquercitrin, rutin, digallic acid, vitexin, D-catechin, quercetin, luteolin, kaempferol, myricetin and so on. The results of the animal experiments showed that MOP down-regulated the expression of MDA and increased the expression of SOD, CAT and GSH-Px. Meanwhile, MOP played a key role by inhibiting the MAPK signaling pathway and activating the Nrf2 signaling pathway during gastric defense and protection. These results provide a substantial contribution to MOP intervention on gastric injury induced by ethanol. Therefore, MOP is expected to provide reference value for the clinical prevention and treatment of gastric injury.

## Data Availability

Data is contained within the article and Supplementary Materials.

## Supplementary Materials

The following supporting information can be downloaded at: https://www.mdpi.com/article/10.3390/antiox11122452/s1, Table S1: Genes and primers for PCR; Table S2: Effects of different resin types on adsorption and desorption of MOP; Table S3 Identification of chemical compounds by UHPLC/Q-TOF-MS/MS.
