# Supplementary material for: Protective Effect of Polyphenols Purified from Mallotus oblongfolius on Ethanol-Induced Gastric Mucosal Injury by Regulating Nrf2 and MAPKs Pathways
Source: Antioxidants (Basel). 2022 Dec 12;11(12):2452. doi: 10.3390/antiox11122452 (PMC9774160; doi:10.3390/antiox11122452)
Supplement: Supplementary file 1 [file antioxidants-11-02452-s001.zip › antioxidants-2048165-supplementary.pdf]

Table S1 Genes and primers for PCR.

Gene	Primer	Accession numbers
**Nrf2**		
**Forward**	5′-GCCTTCCTCTGCTGCCATTAGTC-3′	NM_001399173
**Reverse**	5′-TGCCTTCAGTGTGCTTCTGGTTG-3′	
**HO-1**		
**Forward**	5′-CAGGTGTCCAGGGAAGGCTTTAAG-3′	NM_012580
**Reverse**	5′-TGGGTTCTGCTTGTTTCGCTCTATC-3′	
**Keap1**		
**Forward**	5′-TGCTCAACCGCTTGCTGTATGC-3′	NM_057152
**Reverse**	5′-TCATCCGCCACTCATTCCTCTCC-3′	
**p38**		
**Forward**	5′-GATAAGAGGATCACAGCAGCCCAAG-3′	NM_031020
**Reverse**	5′-TCGTAGGTCAGGCTCTTCCATTCG-3′	
**JNK**		
**Forward**	5′-CCACCACCAAAGATCCCTGACAAG-3′	NM_053829
**Reverse**	5′-GACGCCATTCTTAGTTCGCTCCTC-3′	
**ERK**		
**Forward**	5′-CTGGCTGCTAGGAACATTCTGGTG-3′	NM_001104528
**Reverse**	5′-GTCATCCTGGAGGTAGCGAGAGAG-3′	
**GADPH**		
**Forward**	5′-GACATGCCGCCTGGAGAAAC-3′	NM_017008
**Reverse**	5′-AGCCCAGGATGCCCTTTAGT -3′	

Table S2 Effects of different resin types on adsorption and desorption of MOP. Values with different letters are significantly different ($P < 0.05$).

**Resin type**	**D201**	**AB-8**	**NKA-II**	**NKA-9**	**X-5**
**Moisture content (%)**	50-60%	60-70%	42-52%	65-75%	45-60%
**Partical size （mm）**	0.315-1.25	0.3-1.25	0.3-1.25	0.3-1.25	0.3-1.25
**Category**	negion	low-pole	polarity	polarity	nonpolar
**Adsorption rate (%)**	56.07 ± 0.52^{a}	22.42 ± 0.38^{d}	36.58 ± 0.45^{c}	54.65 ± 0.54^{a}	46.43 ± 0.35^{b}
**Desorption rate (%)**	5.81 ± 0.36^{d}	80.03 ± 1.59^{b}	73.15 ± 1.18^{c}	92.35 ± 1.86^{a}	82.29 ± 1.32^{b}

Table S3 Identification of chemical compounds by UHPLC/Q-TOF-MS/MS.

NO	Retention time(min)	Experimental(m/z) [M+H]+	Theoretical (m/z)	Mass error (mDa)	Fragments (m/z)	Molecular formula	Tentative Identification
**1**	4.81	476.0973	476.0955	1.8	219.0294; 453.0090	$C_{22}H_{20}O_{12}$	Kaempferol 3-O-β-Dglucuronopyranosyl methyl ester
**2**	5.57	318.0739	318.074	-0.1	277.0351	$C_{16}H_{14}O_{7}$	6,8-Dihydroxy-1,2,4-trimethoxyxanthone
**3**	6.06	322.0326	322.0325	0.1	260.0312; 303.0567	$C_{14}H_{10}O_{9}$	Digallic acid
**4**	6.40	530.1764	530.1788	-2.4	329.0662; 415.0297	$C_{27}H_{30}O_{11}$	Icariside-I
**5**	7.01	564.1489	564.1479	1	299.0556; 329.0662; 449.10768	$C_{26}H_{28}O_{14}$	Corymboside
**6**	7.06	318.0379	318.0376	0.3	299.0556	$C_{15}H_{10}O8$	Myricetin
**7**	7.59	580.1433	580.1428	0.5	303.0145; 337.0197	$C_{26}H_{28}O_{15}$	Kaempferol-3-O-β-D-glucoside-7-O-α-L-arabinofuranoside
**8**	8.48	580.1787	580.1792	-0.5	283.0607; 313.0713; 415.1026	$C_{27}H_{32}O_{14}$	Narirutin
**9**	8.59	382.1423	382.1416	0.7	283.0607; 313.0713	$C_{22}H_{22}O_{6}$	Licoricone
**10**	9.39	610.5168	610.5180	-1.2	303.0489; 147. 0632	$C_{27}H_{30}O_{16}$	Rutin
**11**	9.41	302.0432	302.0427	0.6	153.0187; 195.0286	$C_{15}H_{10}O_{7}$	Quercetin
**12**	9.42	465.0955	465.0955	0	303.0502	$C_{21}H_{20}O_{12}$	Isoquercitrin
**13**	9.54	414.095	414.0951	-0.1	313.0712; 397.0922	$C_{21}H_{18}O_{9}$	Pinnatifinoside A
**14**	10.19	432.1061	432.1057	0.4	283.0607; 313.07127	$C_{21}H_{20}O_{10}$	Vitexin
**15**	10.23	464.0956	464.0955	0.1	313.0713; 433.1130	$C_{21}H_{20}O_{12}$	6-Hydroxykaempferol-7-O-glucoside
**16**	11.01	464.0952	464.0955	-0.2	293.0297; 303.0509	$C_{21}H_{20}O_{12}$	Quercetin-3-O-β-D-glucopyranoside
**17**	11.47	286.0482	286.0477	0.5	271.1005	$C_{15}H_{10}O_{6}$	Luteolin
**18**	11.50	422.0822	422.0849	-2.7	219.0292; 247.0242	$C_{19}H_{18}O_{11}$	Isomangiferin

NO	Retention time(min)	Experimental(m/z) [M+H]+	Theoretical (m/z)	Mass error (mDa)	Fragments (m/z)	Molecular formula	Tentative Identification
**19**	12.50	286.0484	286.0477	0.7	287.0558; 331.1004	$C_{21}H_{20}O_{10}$	Kaempferol
**NO**	**Retention time(min)**	**Experimental(m/z) [M+H]+**	**Theoretical (m/z)**	**Mass error (mDa)**	**Fragments (m/z)**	**Molecular formula**	**Tentative Identification**
**20**	13.19	358.1415	358.1416	-0.1	174.067; 219.1018	$C_{20}H_{22}O_6$	3′-O-Angeloylhamaudol
**21**	14.15	340.1314	340.1311	0.4	174.067; 219.1018	$C_{20}H_{20}O_5$	Kushenol S
**22**	14.56	384.1575	384.1573	0.2	219.1018	$C_{22}H_{24}O_6$	Sophoflavescenol
**23**	16.23	624.1702	624.169	1.1	287.0560	$C_{28}H_{32}O_{16}$	Isorhamnetin-3-O-β-rutinoside_1
**24**	16.82	448.1003	448.1006	-0.3	433.1129	$C_{21}H_{20}O_{11}$	Kaempferol-3-O-β-D-glucopyranoside
**25**	20.11	594.1591	594.1585	0.6	433.1131	$C_{27}H_{30}O_{15}$	Kaempferol-3-O-rutinoside
**26**	22.76	676.2366	676.2367	-0.2	153.018; 207.065; 467.0828	$C_{33}H_{40}O_{15}$	Icariin
**27**	23.19	492.0898	492.0904	-0.6	209.154; 331.0453	$C_{22}H_{20}O_{13}$	Quercetin-3-O-glucuronide 6″-methylester
**28**	27.10	290.0795	290.0790	0.5	153.0187	$C_{15}H_{14}O_6$	D-catechin
**29**	28.56	506.1058	506.106	-0.2	285.039; 313.034; 330.037; 345.0608	$C_{23}H_{22}O_{13}$	Quercetin-3-O-(6"-O-acetyl)-β-D-glucopyranoside
**30**	32.08	170.0217	170.0215	0.2	153.0186	$C_7H_6O_5$	Gallic acid
**31**	34.83	500.1661	500.1682	-2.1	277.034; 303.0142	$C_{26}H_{28}O_{10}$	Baohuoside II
**32**	35.16	356.1273	356.126	1.3	153.018; 207.0659	$C_{20}H_{20}O_6$	Leachianone G
**33**	35.26	386.1362	386.1366	-0.4	153.018; 207.0659	$C_{21}H_{22}O_7$	Kushenol W
**34**	35.62	354.1107	354.1103	0.4	153.018; 325.1069	$C_{20}H_{18}O_6$	8-C-Prenylkaempferol
**35**	35.67	344.1264	344.126	0.4	153.0189	$C_{19}H_{20}O_6$	3-(4'-Methoxy-benzyl)-5,7-dihydroxy-6-methyl-8-methoxy-chroman-4-one
**36**	35.70	312.0970	312.0998	-2.8	153.0189	$C_{18}H_{16}O_5$	8-Methoxybonducellin
**37**	35.72	802.2191	802.2168	2.3	467.082; 717.2138	$C_{34}H_{42}O_{22}$	Isorhamnetin-3-gentiobioside-7-glucoside

**38**	35.82	272.0689	272.0685	0.4	181.0501	$C_{20}H_{20}O_6$	Naringenin
**39**	36.72	270.0534	270.0528	0.6	160.0708	$C_{15}H_{10}O_5$	Resokaempferol

**NO**	**Retention time(min)**	**Experimental(m/z) [M+H]+**	**Theoretical (m/z)**	**Mass error (mDa)**	**Fragments (m/z)**	**Molecular formula**	**Tentative Identification**
**40**	39.79	446.1213	446.1213	0	341.0662; 385.0925; 429.11827	$C_{22}H_{22}O_{10}$	3′-Methoxy-5′-hydroxyisoflavone-7-O-β-D-glucoside
**41**	39.81	484.0768	484.0772	-0.4	341.0662; 385.0925	$C_{21}H_{21}O_{11}$	Cyanidin 3-glucoside
**42**	51.49	538.1867	538.1839	2.8	301.1414	$C_{29}H_{30}O_{10}$	Aloeresin G
**43**	55.59	424.2838					Undefined
**44**	58.55	446.1213	446.1181	3.2	281.0517	$C_{22}H_{22}0_{10}$	Rhamnocitrin-3-O-rhamnoside
**45**	59.13	281.2726					Undefined
**46**	62.31	470.2301	470.2305	-0.4	267.1231; 323.1857	$C_{27}H_{34}O_7$	Neokurarinol
**47**	64.50	283.3380					Undefined
**48**	65.18	309.2910					Undefined
**49**	66.19	422.2115	422.2093	2.1	267.0003; 355.0705	$C_{26}H_{30}O_5$	Kushenol U
**50**	67.56	335.3797					Undefined
